# The Role of Toll-like Receptor 2 Polymorphisms in Susceptibility to and Severity of Tuberculosis: A Systematic Review

**DOI:** 10.3390/pathogens15040354

**Published:** 2026-03-27

**Authors:** Sudarto Sudarto, Zen Hafy, Irsan Saleh, Iche Liberty, Zen Ahmad, Fadhyl Zuhry Lubis, Owen Hu, Bryan Arista

**Affiliations:** 1 Division of Pulmonology and Critical Medicine, Department of Internal Medicine, Faculty of Medicine, Universitas Sriwijaya, Dr. Mohammad Hoesin General Hospital, Palembang 30126, Indonesia; sudartokurnia@yahoo.com (S.S.);; 2 Department of Biomedical, Faculty of Medicine, Universitas Sriwijaya, Palembang 30126, Indonesia; 3 Department of Pharmacology, Faculty of Medicine, Universitas Sriwijaya, Palembang 30126, Indonesia; 4 Department of Public Health and Community Medicine, Universitas Sriwijaya, Palembang 30126, Indonesia; 5 Department of Internal Medicine, Faculty of Medicine, Universitas Sriwijaya, Dr. Mohammad Hoesin General Hospital, Palembang 30126, Indonesia

**Keywords:** Toll-like receptor 2, pulmonary tuberculosis, genetic polymorphism, infection susceptibility, disease severity

## Abstract

Pulmonary tuberculosis (TB) remains a global health threat, with individual genetic determinants like Toll-like receptor 2 (TLR2) gene variations potentially modulating immune responses to *Mycobacterium tuberculosis*. This systematic review evaluates the role of TLR2 polymorphisms in influencing susceptibility to and clinical manifestations of pulmonary TB. Following PRISMA guidelines, a comprehensive search of PubMed, Scopus, and ScienceDirect was conducted through July 2024 for observational studies investigating TLR2 single-nucleotide polymorphisms (SNPs) and active TB. Risk of bias was assessed using the Newcastle–Ottawa Scale. Of 8878 identified articles, 32 studies met the inclusion criteria. The most frequently investigated variants, Arg753Gln (rs5743708), −196 to −174 del, and rs3804099, were consistently associated with increased TB susceptibility, particularly in Asian and African populations. Furthermore, specific polymorphisms correlated with greater disease severity, including cavitary lesions and aggressive clinical progression. In conclusion, TLR2 genetic polymorphisms significantly increase the risk of developing pulmonary TB and contribute to more severe clinical outcomes. These findings emphasize the potential of genetic profiling in enhancing TB control strategies and developing personalized diagnostic or therapeutic approaches.

## 1. Introduction

Tuberculosis (TB) is an infectious disease caused by *Mycobacterium tuberculosis* (*M. tb*) and remains a major global public health problem. According to the 2023 WHO report, an estimated 10.6 million new TB cases and approximately 1.3 million deaths occurred worldwide, making TB the second leading cause of infectious mortality after COVID-19 [1]. Indonesia is among the three countries with the highest TB burden globally, alongside India and China [1,2]. The substantial disease burden is driven not only by challenges in diagnosis and access to treatment but also by marked inter-individual heterogeneity in susceptibility to infection and disease progression, which cannot be fully explained by environmental factors alone [3,4,5].

In the immunopathogenesis of TB, the innate immune system plays a crucial role in determining whether exposure to *M. tb* results in latent infection, active TB, or complete clearance of the pathogen [6,7]. One of the key components of innate immunity is the Toll-like receptor (TLR) family, particularly TLR2, which recognizes structural components of the *M. tb* cell wall such as lipoproteins, peptidoglycan, and lipoarabinomannan [8]. Activation of TLR2 triggers MyD88-dependent signalling pathways and induces transcription of proinflammatory cytokines such as TNF-α, IL-6, and IL-12 via NF-κB and MAPK pathways. This immune response activates macrophages and supports Th1-mediated cellular immunity, the principal defence mechanism against *M. tb* infection [8,9,10,11].

However, TLR2 activation exhibits dualistic effects. On the one hand, it contributes to macrophage activation and infection control. On the other hand, chronic TLR2 activation may be exploited by *M. tb* to evade immune elimination [8]. Several studies have shown that TLR2 induces IL-10 expression—an immunoregulatory cytokine—while inhibiting phagosome–lysosome fusion and diminishing antigen presentation by antigen-presenting cells. These phenomena create ambiguity regarding the functional role of TLR2 in TB infection and support the hypothesis that genetic variations within TLR2 may modulate the balance between host resistance and susceptibility to TB [12,13].

Individual genetic factors, particularly polymorphisms within the *TLR2* gene, have been widely investigated as determinants of host susceptibility to TB. Several single nucleotide polymorphisms (SNPs) in *TLR2*—such as rs5743708 (Arg753Gln), rs3804099 (T597C), and the −196 to −174 deletion—are known to alter receptor structure or function, thereby affecting ligand recognition and downstream immune signalling [8]. Epidemiological and molecular studies across different regions have demonstrated associations between specific SNPs and increased risk of active TB, progression from latent to active infection, and more severe clinical phenotypes. However, these findings are often inconsistent across populations due to genetic diversity, ethnic background, and methodological differences [14].

Multiple meta-analyses have reported that rs5743708 is significantly associated with TB susceptibility, particularly in South Asian and North African populations. Conversely, rs3804099 and the −196 to −174 deletion have shown more variable results and, in some populations, no meaningful association. These inconsistencies suggest that the effects of *TLR2* polymorphisms on TB are likely polygenic, complex, and influenced by interactions with environmental factors and the pathogenic characteristics of *M. tb* itself. Accordingly, a systematic review is needed to synthesize existing evidence and critically evaluate the contributions of individual SNPs to TB susceptibility and disease severity [14,15].

Through a systematic review methodology, this study aims to identify, screen, and analyze the scientific evidence from observational studies investigating the association between *TLR2* gene polymorphisms and pulmonary tuberculosis, both in terms of infection susceptibility and disease severity. By focusing on specific genetic variations across human populations, this review seeks to clarify the associations between *TLR2* SNPs and pulmonary TB and to highlight the potential of genetic biomarkers in early detection strategies, risk stratification, and the development of immunomodulatory therapies tailored to individual genetic profiles.

## 2. Materials and Methods

### 2.1. Study Design

This study is a systematic review designed to critically examine and synthesize the available scientific evidence regarding the association between Toll-like receptor 2 (TLR2) genetic polymorphisms and susceptibility to, as well as clinical severity of, pulmonary tuberculosis in human populations. The review protocol was developed in accordance with the Preferred Reporting Items for Systematic Reviews and Meta-Analyses (PRISMA) 2020 guidelines (Table S1) to ensure methodological transparency and rigor. This study does not include quantitative synthesis or meta-analysis; therefore, a descriptive narrative approach was used, tailored to the methodological variability of the included studies [16,17].

### 2.2. Literature Search Strategy

A systematic literature search was conducted across three major electronic databases, PubMed/MEDLINE, ScienceDirect, and Google Scholar, covering publications from each database’s inception to 1 March 2025. The search strategy employed combinations of free-text keywords and Medical Subject Headings (MeSH) terms using Boolean operators (AND, OR) to achieve optimal coverage and sensitivity. Reference management and deduplication were performed using Mendeley Reference Manager version 2.109.0 (Mendeley Ltd., London, UK).

Key search terms included:

- “Toll-like receptor 2” OR “TLR2” OR “TLR2 polymorphism”
- AND “Tuberculosis” OR “Pulmonary tuberculosis” OR “Mycobacterium tuberculosis”
- AND “Genetic susceptibility” OR “Disease severity”
- AND “Single nucleotide polymorphism” OR “SNP”

These terms were applied independently and in multiple combinations. Reference lists of relevant articles were also screened to identify additional studies not captured through database searches. 

### 2.3. Inclusion and Exclusion Criteria

Studies were included if they met the following criteria:

1. Study design: Observational studies (case–control or cohort) evaluating associations between *TLR2* polymorphisms and TB.
2. Population: Human subjects with active or latent pulmonary TB diagnosed using clinical, radiological, or microbiological criteria.
3. Exposure: Studies assessing one or more *TLR2* polymorphisms, such as rs5743708 (Arg753Gln), rs3804099 (T597C), rs5743704, or the −196 to −174 deletion.
4. Outcomes: Occurrence of TB and/or clinical severity (e.g., cavitary lesions, bilateral lung involvement, progression from latent to active TB).
5. Publication type: Full-text articles in Indonesian or English.

Studies were excluded if they:

1. Were case reports, editorials, commentaries, narrative reviews, conference abstracts, or non-human experimental studies.
2. Did not specify the *TLR2* polymorphisms examined.
3. Lacked extractable primary data relevant to the review objectives.

### 2.4. Study Selection Process

Two reviewers independently screened the titles and abstracts of all retrieved articles. Studies deemed potentially eligible were then examined in full text. Discrepancies were resolved through discussion or by consulting a third reviewer when needed. The study selection process is illustrated in a PRISMA flow diagram, outlining the number of records identified, screened, excluded, and included.

### 2.5. Data Extraction and Management

Data were extracted using a standardized form developed in Microsoft Excel version 2402 (Microsoft Corporation, Redmond, WA, USA) using a standardized form capturing: author name, year of publication, study location, study design and sample size (number of cases and controls), population characteristics (ethnicity, age range, TB classification), genotyping methods, specific *TLR2* polymorphisms analyzed, and main outcomes (OR, CI, and statistical significance). Extraction was performed independently by two reviewers and cross-checked for accuracy and consistency. Any discrepancies were resolved through consensus.

### 2.6. Quality Assessment and Risk of Bias

The methodological quality of included studies was assessed using the Newcastle–Ottawa Scale (NOS) for observational studies. The three domains evaluated were: (1) selection of study participants, (2) comparability of groups, and (3) ascertainment of exposure or outcome. Studies scoring ≥7 were classified as high quality, scores 5–6 as moderate, and <5 as low quality. Risk of bias assessment was conducted independently by two reviewers.

### 2.7. Data Synthesis

Given the heterogeneity in study design, population characteristics, SNP variants investigated, and analytical methods, data were synthesized narratively. Findings from individual studies were presented and compared according to the type of *TLR2* polymorphism, direction and strength of association, statistical significance, and geographic or ethnic context of the study populations. The final synthesis is presented through comparative tables and structured narrative summaries to highlight consistent patterns, inter-study discrepancies, and gaps in the current evidence base.

## 3. Results

**Figure 1:**
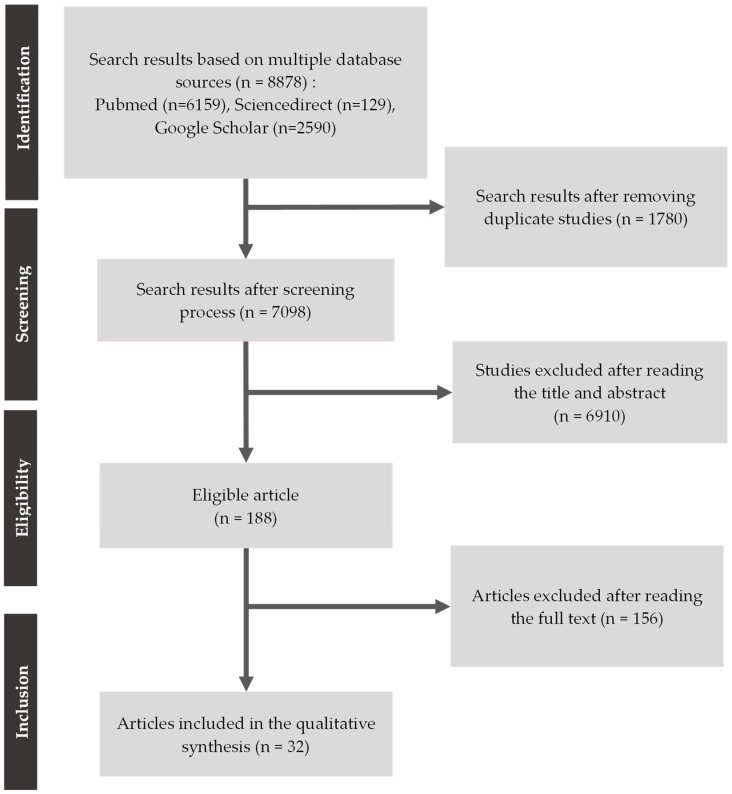
PRISMA flow chart diagram.

The literature selection process followed the PRISMA 2020 guidelines (Figure 1) [16,17]. An initial total of 8878 articles was identified across three electronic databases: PubMed (6159), ScienceDirect (129), and Google Scholar (2590). After removal of 1780 duplicate records, 7098 unique articles remained and were screened based on titles and abstracts. Of these, 6910 articles were excluded for reasons such as not addressing *TLR2* polymorphisms, not involving human subjects, or being editorials or narrative reviews.

A total of 188 articles proceeded to full-text assessment, but 156 were excluded for not meeting the inclusion criteria—for example, failure to specify the *TLR2* SNPs investigated, absence of analyzable primary data, or use of an inappropriate study design. Ultimately, 32 studies met all inclusion criteria and were included in the qualitative synthesis. These comprised observational studies (case–control and cohort) examining the association between one or more TLR2 polymorphisms and susceptibility to, or clinical severity of, pulmonary tuberculosis.

**Figure 2:**
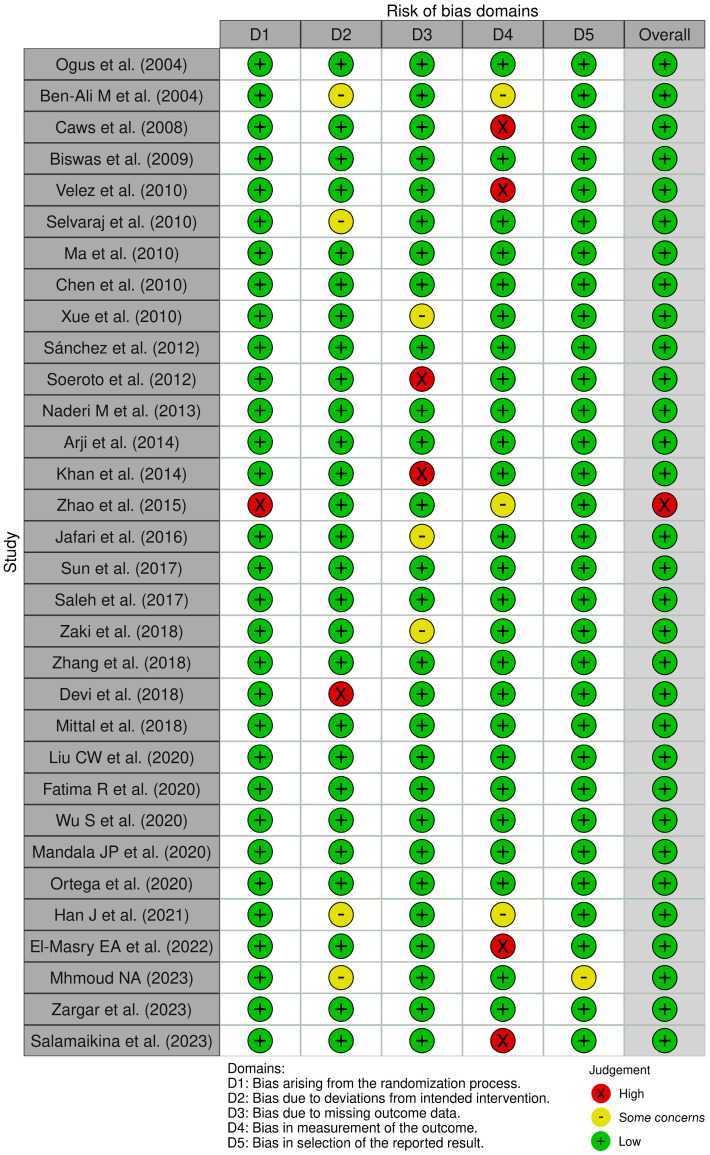
Risk of Bias Assessment Using the Modified Newcastle–Ottawa Scale (NOS) [18,19,20,21,22,23,24,25,26,27,28,29,30,31,32,33,34,35,36,37,38,39,40,41,42,43,44,45,46,47,48,49].

The methodological quality assessment of the 32 included studies using the Newcastle–Ottawa Scale (NOS) (Figure 2) showed that most studies were of good quality. A total of 62.5% were categorized as having an overall low risk of bias, 28% demonstrated a moderate risk (some concerns), and 9.4% exhibited a high risk of bias.

Most studies demonstrated adequate participant selection (D1) and good outcome reporting (D5). However, the risk of bias tended to increase in the domains of outcome measurement (D4) and missing outcome data (D3), particularly in studies with limited methodological detail or without standardized reporting of TB diagnostic criteria. Several studies also did not report adjustment for confounding factors, which may affect the interpretation of internal validity.

Interestingly, no substantial differences in methodological quality were observed between studies from high-income and low- or middle-income countries. Studies from Indonesia, although contributing important data, demonstrated a relatively high risk of bias. These findings highlight the need for improved reporting standards and methodological rigor in genetic studies conducted in the Indonesian population [18]. The principal characteristics and main findings of the 32 included studies are summarized in Table 1.

### Polymorphisms and Clinical Severity of TB

Beyond susceptibility, several SNPs have been investigated in relation to TB clinical severity, including bilateral lung involvement, cavitary lesions, and retreatment. SNPs such as rs3804099 (T597C) and rs76112010 in the study by Zhang et al. (2018) [37] were linked to more severe TB phenotypes and retreatment episodes. These polymorphisms do not consistently correlate with overall TB incidence but appear to influence specific clinical phenotypes, reflecting possible roles in immune modulation or lung tissue pathology [25,50,51].

Similarly, the study by Caws et al. (2008) [21] indicated that the rs3804099 C allele increases susceptibility to TB caused by the Beijing strain of *M. tb*, underscoring the importance of host–pathogen interactions. Findings from Chen et al. (2010) [26] also showed that certain TLR2 genotypes were associated with elevated NK-cell counts and manifestations such as systemic symptoms and pleuritis, providing further evidence that TLR2 polymorphisms may influence not only susceptibility but also disease trajectory.

## 4. Discussion

This systematic review strengthens the evidence that genetic polymorphisms in the *Toll-like receptor 2* (TLR2) gene contribute to host susceptibility and may influence the clinical severity of pulmonary tuberculosis infection. By examining 32 studies across diverse global populations, we identified consistent patterns indicating that TLR2 genetic variation, particularly rs5743708 (Arg753Gln) and the −196 to −174 deletion, plays a meaningful role in modulating immune responses to *Mycobacterium tuberculosis* (*M. tb*). Nonetheless, the magnitude and direction of these effects are not universal and appear to be shaped by ethnic background, environmental context, and gene–gene interactions.

### 4.1. SNP Variants and TB Susceptibility

The rs5743708 (Arg753Gln) SNP is the most extensively studied variant and demonstrates a consistent association with increased TB risk, particularly in South Asian (India, Pakistan) and North African (Tunisia, Sudan) populations. These variant substitutes arginine with glutamine within the ligand-recognition domain of TLR2, impairing MyD88-dependent immune signalling and reducing production of proinflammatory cytokines such as TNF-α and IL-12. Studies by Ogus et al. in Turkey and Ben-Ali et al. in Tunisia reported significant odds ratios (up to 6.04) among individuals carrying the GA or AA genotypes [19,20].

However, the association between rs5743708 and TB susceptibility is not universal. The lack of significant associations observed in several other cohorts. These discrepancies highlight the context-dependent and potentially ethnicity-specific effects of this SNP, reflecting the substantial variation in allele distribution across populations [25,33,45].

In contrast, the −196 to −174 deletion polymorphism, a 22 bp deletion within the TLR2 promoter region, has been shown to reduce gene expression and dampen immune activation against *M. tb*. Studies by Velez et al. (2010) [23], and Khan et al. (2014) [31] in Pakistan, as well as Devi et al. (2018) [38] in India, consistently associated the del/del genotype with increased TB susceptibility, particularly among males and smokers. Conversely, Chen et al. (2010) [26] in Taiwan reported that the same genotype was associated with higher natural killer (NK) cell counts and milder clinical symptoms, suggesting that the biological effects of the promoter deletion may be modulated by epigenetic factors and local innate immune regulation [6,8].

The persistence of distinct ethnic differences in TLR2 polymorphism frequencies, such as the Arg753Gln variant, despite modern globalization and extensive human migration, can be attributed to historical evolutionary pressures. Genetic variations are shaped over millennia by local pathogen-driven selection. For instance, the Arg753Gln substitution, which is present in European populations at frequencies of 3–10%, impairs immune activation but may have conferred evolutionary advantages or protection against other historical endemic pathogens in those regions, such as late-stage Lyme disease or syphilis. Because mass globalization is a relatively recent phenomenon on an evolutionary timescale, these deep-rooted genetic signatures continue to dictate regional and ethnic-specific susceptibility to *M. tb* [52,53].

### 4.2. Gender Differences in TLR2-Mediated Susceptibility

Emerging evidence, including observations by Benbetka et al. (2019) [54], suggests a gender-related role in TLR2-mediated TB susceptibility. Data synthesized in our review supports this hypothesis. Specifically, Khan et al. (2014) [31] demonstrated that the TLR2 promoter deletion (−196 to −174 del) increased TB susceptibility predominantly among male heterozygotes. Similarly, Zhao et al. (2015) [32] reported that the TLR2 rs3804099 polymorphism was associated with an increased risk of pulmonary TB specifically in males. These gender disparities in genetic susceptibility may be driven by the immunomodulatory effects of sex hormones on TLR expression and downstream innate immune responses, highlighting the need to stratify future genetic association studies by sex.

### 4.3. Evidence from Indonesia: Limited but Clinically Relevant

One of the few studies assessing TLR2 polymorphisms in Indonesia, conducted by Soeroto et al. (2018) [18], reported that the Arg753Gln variant was associated with a markedly increased risk of active TB (OR 28.07; *p* = 0.022), though it did not alter cytokine levels such as IFN-γ, TNF-α, or IL-10. This suggests that the pathogenic mechanism may be driven more by structural alterations in the receptor than by systemic cytokine modulation. However, genetic data from Indonesia remain sparse and do not yet represent the country’s extensive ethnic diversity.

Given that Indonesia ranks among the top three countries globally in TB burden, expanding genetic studies is essential for identifying genetically high-risk populations and developing locally tailored screening strategies and immunotherapeutic approaches.

### 4.4. Clinical Implications and Future Directions

The findings of this review support TLR2 polymorphisms particularly rs5743708 and the −196 to −174 deletion as promising genetic biomarkers for identifying populations at increased risk of TB. However, clinical implementation requires cross-population validation and integration of additional data, including epigenetic profiles, host immune status, and local *M. tb* strain characteristics. Certain SNPs such as rs3804099 and specific haplotype combinations (e.g., rs3804099–rs3804100) also show potential as prognostic markers for severe or recurrent TB, rather than for primary susceptibility [8,55,56].

The main limitations across the reviewed studies include the inherent biases of observational designs, insufficient adjustment for confounders, heterogeneity in genotyping methods, and inconsistent definitions of TB phenotypes. Therefore, large-scale, multi-ethnic studies with standardized diagnostic and genotyping methods are needed. Integrative approaches using advanced genomic technologies such as whole-genome sequencing and functional pathway analysis are strongly recommended to provide more definitive insights into the roles of TLR2 SNPs.

## 5. Conclusions

This systematic review demonstrates that genetic polymorphisms in the TLR2 gene particularly rs5743708 (Arg753Gln) and the −196 to −174 deletion play important roles in increasing susceptibility to pulmonary tuberculosis and, in certain cases, contribute to more severe clinical manifestations. These associations are more consistent in specific populations, such as those in South Asia and North Africa, but vary across other regions including East Asia and Latin America, reflecting underlying ethnic and environmental influences on gene expression. Additionally, some variants such as rs3804099, although not consistently associated with TB incidence, show potential as markers of more severe or recurrent disease. In Indonesia, although data remain limited, existing studies indicate that TLR2 polymorphisms may also influence TB susceptibility in local populations, underscoring the need for broader, more representative research particularly given the country’s high TB burden and ethnic diversity. Overall, the findings support the potential of TLR2 as a genetic biomarker for TB risk stratification. Nonetheless, validation through large-scale, multi-ethnic studies and functional genomic approaches is required before broad clinical implementation can be achieved.

## Figures and Tables

**Table 1:** Summary of Studies Evaluating the Association Between TLR2 Polymorphisms and Susceptibility, Severity, and Resistance to Tuberculosis Infection.

No.	Authors (Year)	Country	SNP	Study Focus	Sample Size (Cases/ Controls)	Genotyping Method	Clinical Description
1	Ogus et al. (2004) [19]	Turkey	Arg753Gln (rs5743708)	TB Susceptibility	151/116	PCR and sequencing	The frequency of the A (Gln) allele was higher among TB patients (17.9%) compared with controls (7.7%). The AA genotype conferred a 6.04-fold increased risk of TB, while the GA genotype conferred a 1.60-fold increased risk compared with GG, indicating that the Arg753Gln variant is significantly associated with increased susceptibility to tuberculosis in the Turkish population.
2	Ben-Ali M et al. (2004) [20]	Tunisia	Arg677Trp	TB Susceptibility	132/110	Arg677Trp genotyping	The Arg677Trp variant was identified in 94% of TB patients compared with 31% of controls; this variant significantly increased TB risk in the Tunisian population (*p* < 0.0001).
3	Caws et al. (2008) [21]	Vietnam	T597C	TB Susceptibility/Degree of TB Severity	237/392	PCR and sequencing	The C allele of the TLR2 T597C polymorphism was significantly associated with increased risk of TB caused by the *Mycobacterium tuberculosis* Beijing strain, suggesting an interaction between host genotype and bacterial lineage.
4	Biswas et al. (2009) [22]	India	Arg753Gln, Arg677Trp, and Phe749Tyr (novel)	Susceptibility to Lung TB	100/100	PCR-RFLP + DNA sequencing	No Arg753Gln or Arg677Trp SNPs were detected in either TB patients or controls, indicating no association between these polymorphisms and TB in the Indian population. A novel polymorphism, Phe749Tyr, was identified in two patients; however, it did not significantly alter the structure or functional activity of the TLR2 protein. This study supports the presence of ethnic variation in the distribution of TLR2 SNPs.
5	Velez et al. (2010) [23]	Pakistan	−196 to −174 del	TB Susceptibility	306/354	PCR	A promoter deletion in TLR2 was found to increase TB risk in the Pakistani population.
6	Selvaraj et al. (2010) [24]	India	Arg753Gln (2258G/A)	TB Susceptibility	206/212	PCR-RFLP	No significant differences were observed in allele or genotype frequencies of the TLR2 Arg753Gln polymorphism between pulmonary TB patients and healthy controls, and no meaningful association was identified.
7	Ma et al. (2010) [25]	China	Arg753Gln (2258A/G)	TB Susceptibility/Degree of TB Severity	543/544	PCR-RFLP	No significant association was found between the TLR2 Arg753Gln polymorphism and susceptibility to pulmonary TB in the Han Chinese population. The TLR2 (Arg753Gln) variant was also not statistically significant in determining the severity of pulmonary tuberculosis in this population.
8	Chen et al. (2010) [26]	Taiwan	−100 (GT repeats), −16934T > A, −15607A > G, −196 to −174 ins/del, 1350T > C	TB Susceptibility/Degree of TB Severity	184/184	PCR and sequencing	An association was identified between the haplotype [A-G-(insertion)-T] and increased susceptibility to pulmonary TB. The del/del genotype at the −196 to −174 locus was less frequent among patients with systemic symptoms and was associated with higher circulating NK-cell counts. The 1350 CC genotype was more commonly observed in patients with pleuritis and was likewise linked to elevated NK-cell numbers. A homozygous GT short-repeat pattern was also associated with increased NK-cell levels. These findings indicate that TLR2 genetic variations influence TB susceptibility and modulate clinical phenotypes, particularly through regulation of NK-cell expansion.
9	Xue et al. (2010) [27]	China	Arg677Trp, Arg753Gln	TB Susceptibility	205/203	PCR & DNA sequencing	No Arg677Trp or Arg753Gln variants were detected in this population sample. These results suggest that TLR2 SNPs do not significantly contribute to pulmonary TB susceptibility in the Han Zhejiang population of China.
10	Sánchez et al. (2012) [28]	Colombia	Arg753Gln	TB Susceptibility	94/178	PCR and sequencing	No significant association was identified between the TLR2 Arg753Gln SNP and pulmonary TB.
11	Soeroto et al. (2018) [18]	Indonesia	Arg753Gln, Arg677Trp	TB Susceptibility	86/latent TB (quantity unknown)	PCR-RFLP	The TLR2 Arg753Gln polymorphism increased the risk of active TB (OR 28.07, *p* = 0.022), but did not affect levels of IFN-γ, TNF-α, IL-10, or IL-12; Arg677Trp was not detected.
12	Naderi M et al. (2016) [29]	Iran	TLR1 rs5743551, rs5743618	TB Susceptibility	203/203	PCR-RFLP	The G allele of rs5743618 was associated with an increased risk of pulmonary TB in the Iranian population.
13	Arji et al. (2014) [30]	Morocco	TLR2 + 597T/C, −16934T/C, +1349T/C	TB Susceptibility	343/203	PCR-RFLP and TaqMan SNP genotyping	The TLR2 +597CT genotype demonstrated a protective effect against pulmonary TB (OR = 0.65, *p* = 0.04). The TLR2 haplotype [−16934T, +597C, +1349T] showed an increased risk (OR = 1.52), although the association was not statistically significant. An interaction between TLR2 +597 and TLR4 +4434 conferred a combined protective effect.
14	Khan et al. (2014) [31]	Pakistan	TLR2 (–196 to –174del)	TB Susceptibility	87/100	PCR	The del/del genotype was more frequently observed in TB patients than in healthy controls. This polymorphism increased TB susceptibility, particularly among male heterozygotes (I/D), individuals aged 21–45 years, and smokers.
15	Zhao et al. (2015) [32]	China	rs3804099 (T597C)	TB Susceptibility	341/386	PCR-RFLP	The TLR2 597CC genotype (rs3804099) was associated with increased risk of pulmonary TB, especially in males. No significant association was found with tuberculous meningitis (TBM).
16	Jafari et al. (2016) [33]	Iran	Arg753Gln (rs5743708)	TB Susceptibility	96/122	PCR–RFLP	The rs5743708 (Arg753Gln) variant did not show a significant association with susceptibility to pulmonary tuberculosis in the Iranian population.
17	Sun et al. (2017) [34]	China	rs5743708, rs3804099	TB Susceptibility	214/205	PCR and SNaPshot minisequencing	No significant association was found between the two SNPs and TB susceptibility; the CT genotype of rs5743708 was slightly more frequent in TB patients, but the difference was not statistically significant.
18	Saleh et al. (2017) [35]	Egypt	Arg753Gln (753G > A), Arg677Trp (677C > T)	TB Susceptibility	96/50	PCR–RFLP	The GA genotype of Arg753Gln was significantly associated with an increased risk of pulmonary TB (OR 4.83) and peritoneal TB (OR 6.2), whereas Arg677Trp showed no meaningful association.
19	Zaki et al. (2018) [36]	Sudan	rs1816702, rs3804099, rs7656411	TB Susceptibility	207/395	PCR-RFLP (tag SNPs selected)	The TLR2 rs7656411 polymorphism demonstrated a significant association with susceptibility to active pulmonary tuberculosis with individuals carrying the TG and GG genotypes exhibiting a markedly increased risk. In contrast, rs1816702 and rs3804099 showed no significant relationship with TB occurrence, suggesting that these SNPs are unlikely to contribute to genetic susceptibility to active tuberculosis disease in this population.
20	Zhang et al. (2018) [37]	China	rs3804099, rs3804100, rs76112010	TB Susceptibility/Degree of TB Severity	634/475	2 × 48-Plex SNP-scan™ custom assay	No significant differences were observed for the three SNPs between TB cases and controls overall; however, rs3804099 was significantly associated with increased risk in the subgroup of TB patients undergoing retreatment, and rs76112010 was correlated with hematologic parameters such as erythrocyte count, hemoglobin, and hematocrit (p).
21	Devi et al. (2018) [38]	India	TLR2Δ22 (−196–174, 22 bp deletion)	TB Susceptibility	169/227	PCR-RFLP + DNA sequencing	A 22 bp deletion in the TLR2 promoter increased the risk of pulmonary TB, particularly when combined with susceptible genotypes of the VDR gene (BsmI *b/b* or FokI *F/f*).
22	Mittal et al. (2018) [39]	India	−196 to −174del; Arg753Gln (rs5743708)	TB Susceptibility	100/130	Allele-specific PCR (del); PCR-RFLP (Arg753Gln)	The −196 to −174del polymorphism in TLR2 was significantly associated with drug-resistant TB, particularly XDR-TB, while the Arg753Gln SNP showed no significant association.
23	Liu CW et al. (2020) [40]	Taiwan	rs3804099-rs3804100 haplotype	TB Susceptibility	230/213	PCR-RFLP	The C–T haplotype was associated with an increased risk of TB (*adjusted OR ≈ 3.5*).
24	Fatima R et al. (2020) [41]	Pakistan	Arg753Gln	TB Susceptibility	122/122	PCR-RFLP	This study found that the TLR2 Arg753Gln polymorphism was associated with increased susceptibility to tuberculosis in the Pakhtun population of Pakistan.
25	Wu S et al. (2020) [42]	China	rs1898830 TLR2	TB Susceptibility	636/608	PCR-RFLP	The study reported that the rs1898830 genetic variant of the Toll-like receptor 2 (TLR2) gene showed a significant association with tuberculosis susceptibility in two major ethnic groups in China, namely the Han and Tibetan populations.
26	Mandala JP et al. (2020) [43]	India	2258G > A, 2029C > T	TB Susceptibility	102/102	PCR-RFLP	The TLR2 2029C > T polymorphism demonstrated a strong and significant association with tuberculosis susceptibility across co-dominant, dominant, and over-dominant models. Individuals with the C/T genotype had a higher risk, whereas the T/T genotype was protective. For TLR2 2258G>A, a weak association was observed only in the over-dominant model. These findings highlight the important role of TLR2 in the immune response to TB, particularly the 2029C>T variant.
27	Ortega et al. (2020) [44]	Mexico	rs5743708 (R753Q)	TB Susceptibility	279/569	PCR-RFLP	No significant association was found between the TLR2 rs5743708 (R753Q) polymorphism and active TB in this population.
28	Han J et al. (2021) [45]	Taiwan	rs3804100, rs3804099	TB Susceptibility	197/217	PCR-RFLP	No significant association was identified between the TLR2 rs3804100 and rs3804099 SNPs and tuberculosis susceptibility in the Mongolian population.
29	El-Masry EA et al. (2022) [46]	Egypt	Arg753Gln	TB Susceptibility	52/50	PCR-RFLP	The GA genotype (Arg753Gln) was more frequently observed in patients with pulmonary TB and showed a significant association.
30	Mhmoud NA (2023) [47]	Sudan	rs5743557, rs4833095, rs5743708	TB Susceptibility	160/220	PCR-RFLP	Several SNPs (rs5743557, rs4833095, rs5743708) were significantly associated with pulmonary tuberculosis (PTB) in case groups.
31	Zargar et al. (2024) [48]	India	rs4833095, rs5743708	TB Susceptibility	150/150	PCR-RFLP	The frequency of TLR2 polymorphisms was high (73.9%) among patients younger than 50 years. A difference in genotype distribution was observed between cases and controls, supporting the role of TLR2 in TB susceptibility.
32	Salamaikina et al. (2023) [49]	East Europe & Middle East Asia	rs5743708 (exon) & rs3804100	TB Susceptibility/Degree of TB Severity	178/201	Genotyping PCR real-time & epigenetic expression/registration	No significant association was found between the rs5743708 (TLR2) SNP and TB susceptibility after multiple testing correction. However, there was a trend toward the GG genotype being associated with a more rapid decline in CD4+ T-cell count, which may indicate a potential adverse impact on immunological status, although not directly linked to clinical severity of TB.

## Data Availability

No new data were created or analyzed in this study. Data sharing is not applicable to this article.

## Supplementary Materials

The following supporting information can be downloaded at: https://www.mdpi.com/article/10.3390/pathogens15040354/s1, Table S1: PRISMA 2020 Checklist.

## Abbreviations

The following abbreviations are used in this manuscript:

TB	Tuberculosis
TLR2	Toll-like receptor 2
*M. tb*	*Mycobacterium tuberculosis*
SNP	Single nucleotide polymorphism
PRISMA	Preferred Reporting Items for Systematic Reviews and Meta-Analyses
WHO	World Health Organization
COVID-19	Coronavirus disease 2019
TLR	Toll-like receptor
MyD88	Myeloid differentiation primary response 88
NF-κB	Nuclear factor kappa-light-chain-enhancer of activated B cells
MAPK	Mitogen-activated protein kinase
TNF-α	Tumor necrosis factor-alpha
IL-6	Interleukin-6
IL-10	Interleukin-10
IL-12	Interleukin-12
Th1	T helper 1
PCR	Polymerase chain reaction
